# Energy evolution and catastrophic instability of excavated consequent slopes: A cusp catastrophe-based criterion and gradient-anchoring reinforcement strategy

**DOI:** 10.1371/journal.pone.0354142

**Published:** 2026-08-20

**Authors:** Zhaofeng Liu, Bin Du, Huadong Zhu, Yonglei Jiang, Tengwen Wang, Wei Chen, Yanlin Zhao, Yuanzeng Wang

**Affiliations:** 1 School of Smart Construction and Energy Engineering, Hunan Institute of Engineering, Xiangtan, China; 2 College of Civil Engineering, Guizhou University, Guiyang, China; 3 Guizhou Shunkang Testing Co., Ltd., Guiyang, China; 4 School of Resource, Environment and Safety Engineering, Hunan University of Science and Technology, Xiangtan, China; China Construction Fourth Engineering Division Corp. Ltd, CHINA

## Abstract

Understanding energy evolution and associated entropy production during slope excavation is crucial for predicting catastrophic failures in geomechanical systems. This study, from the perspective of energy conservation, investigated the stability of a phosphorus mine tailings dam in Guizhou. By integrating cusp catastrophe theory, we establish a novel energy catastrophe instability criterion, defining the characteristic value Δ as a dynamic indicator of system stability, where Δ > 0 denotes a stable state and Δ < 0 indicates an unstable state. The reliability of this criterion is rigorously validated by comparing the evolution of Δ with the development patterns of the plastic zone across sequential excavation stages (0–50 m depth). Results demonstrate a critical transition: slopes remain stable (Δ > 0) during excavation to 40 m depth but become unstable (Δ < 0) upon reaching 50 m. Crucially, leveraging insights from the energy catastrophe analysis (specifically the spatial and temporal evolution characteristics of system instability precursors), a gradient prestressed anchor cable support strategy was designed and implemented. Field monitoring data confirms the efficacy of this energy-informed reinforcement, showing significantly enhanced slope stability with Δ values persistently maintained in the positive regime.

## 1. Introduction

Slope stability in open-pit mines is a critical issue for safe production [1–5]. Slopes can be classified into consequent, reverse, and transverse slopes based on the relationship between the rock layer strike and slope strike, and further categorized into gentle-dip and steep-dip slopes according to the rock layer dip angle [6–9]. Gentle-dip consequent slopes, characterized by parallel strikes and identical dip directions between rock layers and slopes, exhibit low interlayer friction due to their small dip angles (typically 10°–30°), leading to potential sliding along bedding planes. Continuous mining in open-pit environments exacerbates instability risks for consequent slopes due to complex geological structures, heterogeneous rock strength, and excavation disturbances, often resulting in catastrophic landslides with significant economic losses and safety hazards [10–14]. The Wengfu Phosphate Mine in Guizhou Province, a typical open pit mine, features consequent slope structures significantly affected by weathering and excavation. Establishing a scientific stability evaluation system for such slopes is urgently required.

Extensive research has been conducted on the stability of layered slopes, yielding significant insights. Feng et al. [15] conducted comparative analyses of vibration tests and numerical simulations to investigate slope failure mechanisms, revealing that bedding rock slopes predominantly fail through tensile-shear sliding, with surface dynamic responses significantly exceeding internal responses. Mahmoodzadeh et al. [16] employed machine learning to evaluate the safety factor (FS) of rock slopes, validating model accuracy using Janbu’s limit equilibrium method and GeoStudio. Their results demonstrated that the random forest model achieved the highest accuracy in FS estimation. Liu et al. [17] analyzed landslide characteristics and stability of bedding rock slopes in the Sijiaying Open-Pit Mine. By integrating field investigations, mechanical mechanisms, and reinforcement principles, they identified intersecting bedding planes, weak interlayers, and slope-bedding intersections as intrinsic geological triggers for landslides, with blasting vibrations and rainfall acting as external factors. A stability control strategy derived from these findings significantly enhanced slope stability. Zhou et al. [18] systematically analyzed the energy evolution law of open-pit slope, and proposed a dynamic instability criterion based on cusp catastrophe theory considering the whole slope, which provides a new method for mine slope early warning. Li et al. [19] developed a cusp catastrophe model for stratified steep-dip bedding slopes under excavation disturbances. Experimental results indicated that increased stratum thickness enhances bending stiffness, whereas greater dip angles reduce critical slope length. When dip angles exceed 40°, the critical slope length increases caused by thicker strata stabilizing. Deressa et al. [20] established a numerical model under combined static-dynamic conditions to quantify the effects of blasting design and bench geometry on slope stability using the strength reduction method. Alejano et al. [21] identified critical points in catastrophe theory as instability thresholds, determining through FS that instability transitions occur within FS = 1.61–0.92.

While traditional limit equilibrium and numerical methods partially elucidate slope failure mechanisms, they inadequately describe the nonlinear dynamics of energy evolution and catastrophic instability [21–26]. Energy catastrophe theory, which analyzes critical energy accumulation-dissipation states, provides a novel perspective for early warning of instability, holding both theoretical and practical value. However, existing energy catastrophe models often overlook the influence of complex geological structures (e.g., weathered rock masses, consequent slopes) in open-pit mines. To address this, this study establishes a discriminant Formula for energy catastrophe during excavation of consequent slopes, integrating cusp catastrophe theory and energy conservation principles. The energy catastrophe characteristic value Δ is introduced as a dynamic instability criterion. Through secondary development of FLAC3D, a dissipative energy evolution model was embedded into numerical simulations, enabling multidimensional validation by comparing Δ with plastic zone evolution. Furthermore, a “gradient prestressed anchor cable support” technique was proposed based on energy instability mechanisms. Field trials confirmed the structure’s efficacy in absorbing dissipative energy, providing a theoretical foundation and empirical data for stability control in open pit mine slopes.

## 2. Project description and geological conditions

### 2.1. Project description

**Fig 1:**
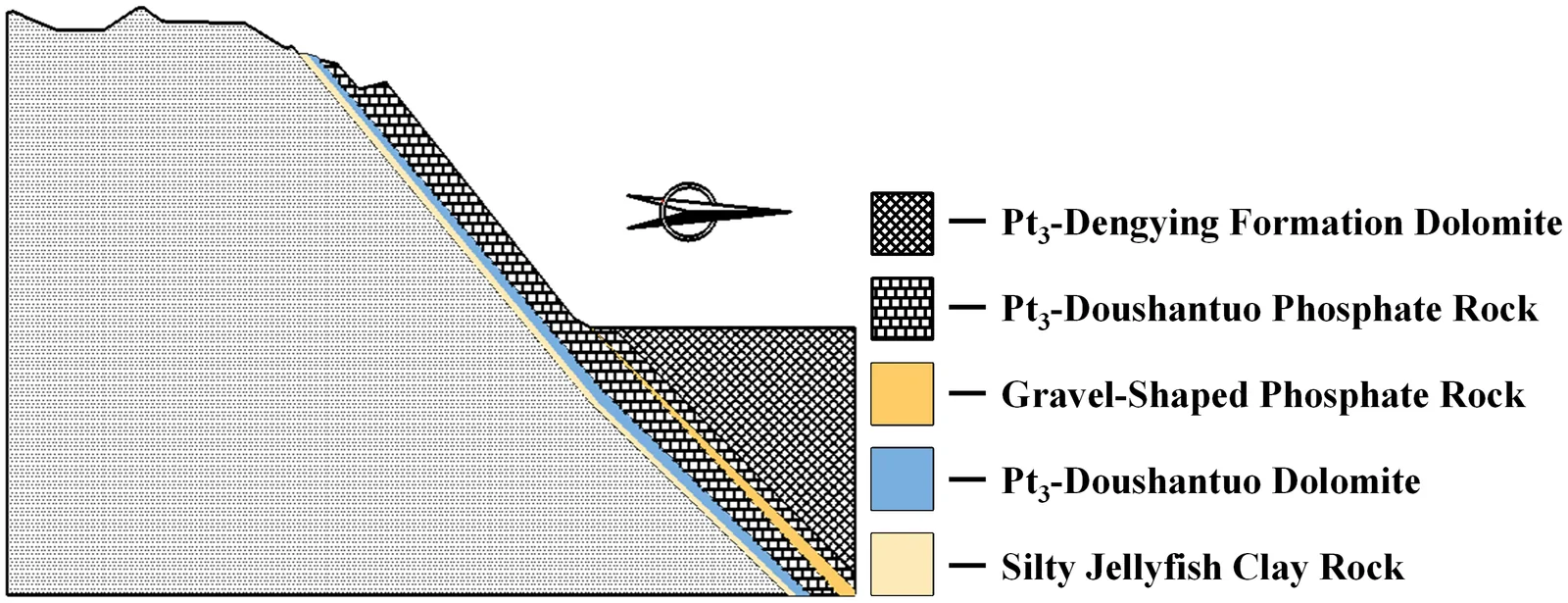
Full-view characteristics of the consequent slope formed in the open-pit.

The open pit mine is in Guizhou Province. Based on current mining depth, the western wing floor of the open-pit forms an engineered slope with a length of 350 m, height of 100–120 m, slope direction of 100°, and gradient of 30°–50°. The slope primarily comprises consequent rock masses composed of siliceous dolomite, claystone, and conglomerate. The upper section is covered by unevenly distributed slag fill (0–40 m thick) and landslide deposits (Fig 1). The current mining elevation is 1180 m, with the designed lowest mining elevation at 1090 m. Continued excavation will result in a final engineered slope height of 170–220 m in the western wing.

The geological data presented in this study, including excavation depth and strata dip angle, were obtained through field surveys and borehole drilling. Measurement uncertainties arise primarily from topographical variations and drilling deviations. The excavation depth measurements have an estimated error of ±0.5 m based on the precision of the surveying equipment used. The strata dip angles, determined from core samples and borehole televiewer data, carry an uncertainty of approximately ±2° due to local structural variations and core orientation limitations. These uncertainties are considered acceptable for the scale of slope stability analysis conducted herein, and their potential influence on the calculated Δ values is discussed in Section 4.2.

### 2.2. Stratigraphic properties

**Fig 2:**
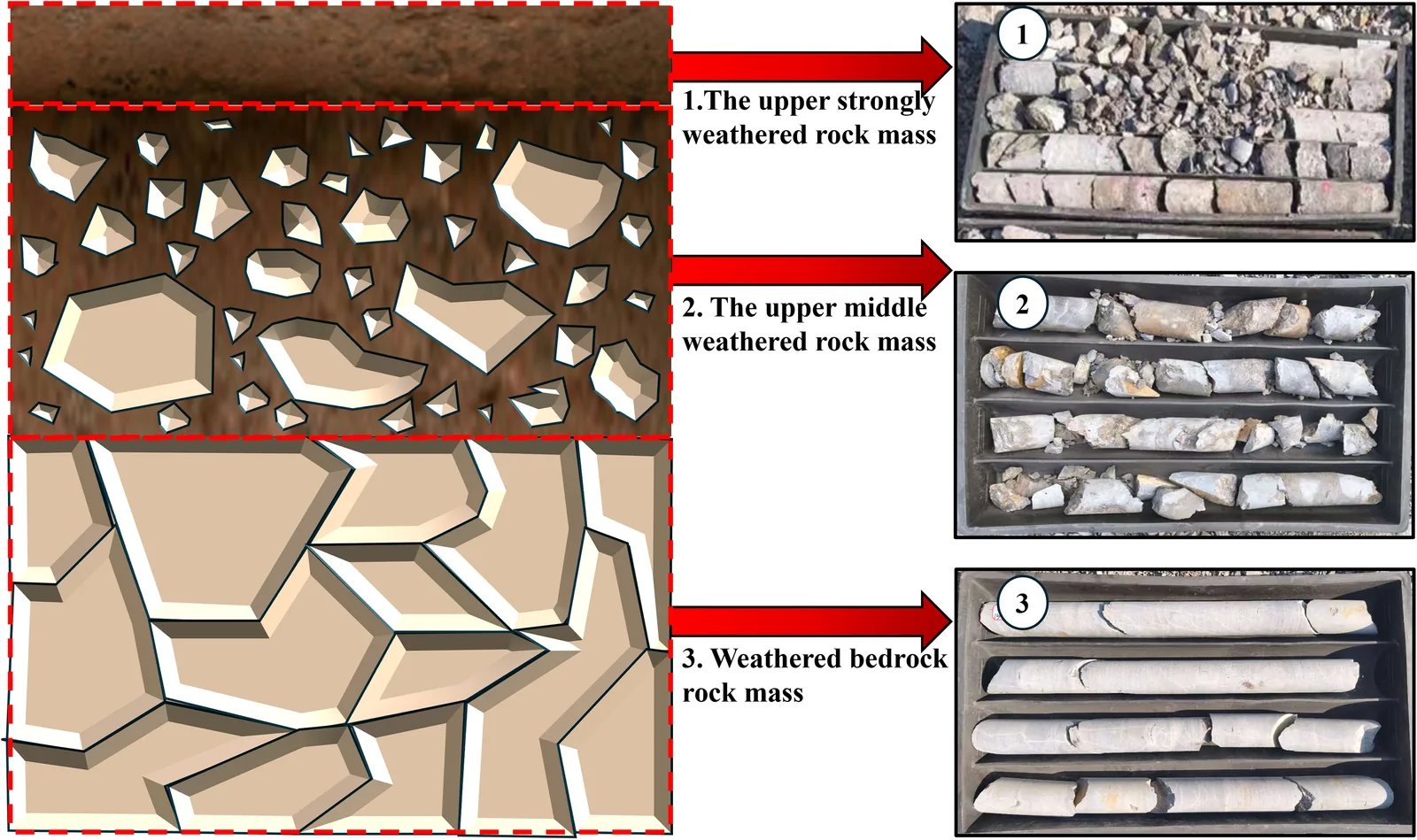
Drill core samples from the exploration line.

Geological drilling data reveal significant weathering and fragmentation in the bedrock zone of the slope. The upper biotite granulite layer is divided into two geotechnical units based on weathering intensity: Highly weathered zone: Extremely fractured rock mass structure. Moderately weathered zone: Partially fragmented rock mass (Fig 2). With increasing depth, the rock transitions to fresh biotite granulite, characterized by intact structure and stable bearing capacity, forming a reliable foundation layer.

Physical and mechanical parameters of the slope’s lithologic layers were systematically determined through field sampling, laboratory testing, and geotechnical investigations (Table 1). To convert rock block shear strength indices to rock mass parameters, a differential reduction method was applied: To convert rock block shear strength indices to rock mass parameters, a differential reduction method was applied based on the Geological Strength Index (GSI) system and the rock mass integrity evaluation [27–29]. Given the highly fractured nature of the rock mass (GSI ≈ 25 ~ 35) as indicated by the drill core observations (Fig 2), the reduction factors were determined as follows: the cohesion was reduced to 5% of its intact value, while the internal friction angle retained 80% of the intact value. This differential treatment reflects the fact that cohesion is more severely degraded by fracturing and weathering than friction, which is consistent with the empirical correlations established by Zhao et al. [28] for disturbed rock masses. The specific values of 0.05 and 0.8 were calibrated against the laboratory test results and the observed field performance of the slope. Specifically, the cohesion of rock blocks was reduced to 5% of its original value, while the internal friction angle retained 80% of the intact rock value.

**Table 1:** Physical and mechanical parameters of key lithologic layers.

Lithology	Gravity γ/kN*m3	Average cohesion of the test c/MPa	Average internal friction angle of the test φ/°	Reduced cohesion c/MPa	Reduced internal friction angle φ/°
Siliceous Dolomite	26.4	1.376	49.2	68.8	39.4
Glacial Till Conglomerate (Saturated)	25.7	0.986	39.0	49.3	31.2
Silty Hydromica Clay Rock (Saturated)	21.0	0.769	37.4	38.4	29.9
Tuffaceous Siltstone	26.0	3.562	47.4	178.1	37.9

## 3. Catastrophic energy instability criterion for excavation of consequent slopes

### 3.1. Cusp catastrophe theory

Catastrophe theory [3,30,31], derived from topology, singularity theory, and topological dynamics, investigates discontinuous changes and abrupt transitions in systems. It describes the evolution of a system from one stable state to another under varying parameters, providing a framework to quantify discontinuous phenomena. In geotechnical engineering, cusp catastrophe theory has been widely adopted to model unpredictable geological issues through empirical or analytical approaches [32–34].

**Fig 3:**
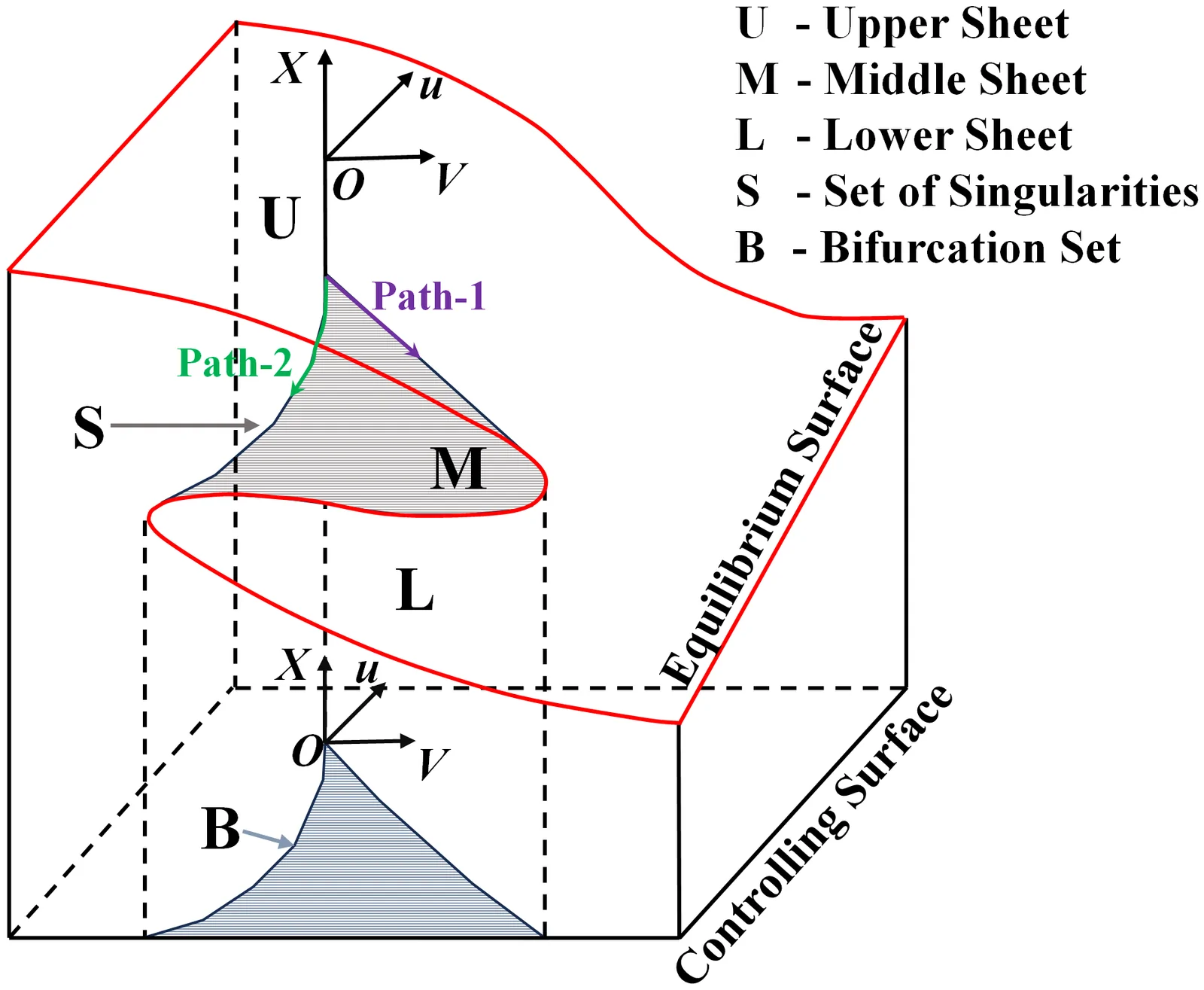
Schematic diagram of the cusp catastrophe model.

Among catastrophe models (cusp, swallowtail, and butterfly), the catastrophe model (Fig 3) is most prevalent due to its tractable critical surface construction [35–37].

The potential function of cusp catastrophe theory is expressed as:


$$\begin{array}{c} V\left(\text{x}\right)=\frac{\text{1}}{\text{4}}x^{\text{4}}+\frac{\text{1}}{\text{2}}\alpha x^{\text{2}}+\beta x \end{array}$$ (1)

The equilibrium surface and singularity set Formulas are derived as:


$$\begin{array}{c} \begin{array}{c} V^{\prime }\left(x\right)=x^{\text{3}}+\alpha x+\beta =0\\ V^{\prime \prime }\left(x\right)=3x^{\text{2}}+\alpha =0 \end{array} \end{array}$$ (2)

By solving Formulas (1) and (2), the bifurcation set Formula is obtained:


$$\begin{array}{c} \Delta =4\alpha^{\text{3}}+27\beta^{\text{2}} \end{array}$$ (3)

The equilibrium surface represents critical states of the system. The instability criterion is defined by crossing the bifurcation set (Δ = 0). When control variables *α* and *β* satisfy Formula (3), the system reaches a critical instability threshold.

### 3.2. Energy evolution characteristics during excavation of consequent slopes

Under external actions (e.g., gravity, temperature), slopes deform and gradually transition from stable to unstable states [38,39]. According to the first law of thermodynamics, the total energy of the slope system remains constant in a dynamic equilibrium energy field, where external actions act as energy inputs and slope deformation serves as energy outputs. The total energy *U* input into the slope system is partitioned into three components: Elastic strain energy (Δ*U*_e_): Stored in the slope rock mass as elastic deformation. Dissipative energy (Δ*U*_*d*_): Released through internal damage, heat exchange, and electromagnetic radiation during sliding. Kinetic energy (Δ*U*_*k*_): Manifests as macroscopic sliding failure of the rock mass [40–44].

Since energy inputs from external loads, temperature, and electromagnetic radiation are negligible compared to gravitational work, these terms are omitted in the energy analysis. Thus, during numerical simulations, the total energy input to the slope system is approximated as the gravitational work (*U* = Δ*U*_*g*_). The energy conservation Formula simplifies to:


$$\begin{array}{c} \Delta U_{g}=\Delta U_{e}+\Delta U_{d}+\Delta U_{k} \end{array}$$ (4)

where: Δ*U*_*g*_: Mechanical work by self-weight stress (gravitational potential energy reduction), defined as the decrease in gravitational potential energy after deformation relative to the initial state.; *ΔU*_e_, Δ*U*_*d*_ and Δ*U*_*k*_: Increments of elastic strain energy, dissipative energy, and kinetic energy during deformation and instability.

For an arbitrary 3D grid element in the slope system under triaxial stress (*σ*_1_, *σ*_2_, *σ*_3_), the gravitational potential energy density *u*_*g*_, elastic strain energy density *u*_*e*_, and kinetic energy density *u*_*k*_ are expressed as:


$$\begin{array}{c} u_{g}=\rho gh \end{array}$$ (5)

$$\begin{array}{c} u_{e}=\frac{\text{1}}{2E}\left[{\sigma_{\text{1}}}^{\text{2}}+{\sigma_{\text{2}}}^{\text{2}}+{\sigma_{\text{3}}}^{\text{2}}-2\upsilon \left(\sigma_{\text{1}}\sigma_{\text{2}}+\sigma_{\text{1}}\sigma_{\text{3}}+\sigma_{\text{2}}\sigma_{\text{3}}\right)\right] \end{array}$$ (6)

$$\begin{array}{c} u_{k}=\frac{\text{1}}{\text{2}}\rho v^{\text{2}} \end{array}$$ (7)

Where: *σ*_1_, *σ*_2_, *σ*_3_: Principal stresses. *E*: Elastic modulus. *ρ*: Density. *υ*: Poisson’s ratio. *v*: Centroid velocity. *h*: Element height.

The total dissipative energy Δ*U*_*d*_ is calculated as:


$$\begin{array}{c} \Delta U_{d}=\rho gh-\frac{\text{1}}{2E}\left[{\sigma_{\text{1}}}^{\text{2}}+{\sigma_{\text{2}}}^{\text{2}}+{\sigma_{\text{3}}}^{\text{2}}-2\upsilon \left(\sigma_{\text{1}}\sigma_{\text{2}}+\sigma_{\text{1}}\sigma_{\text{3}}+\sigma_{\text{2}}\sigma_{\text{3}}\right)\right]-\frac{\text{1}}{\text{2}}\rho v^{\text{2}} \end{array}$$ (8)

Where Δ*U*_*d*_ encompasses both plastic dissipation (irreversible deformation due to plastic flow) and damage-related energy dissipation (energy consumed by microcrack initiation, propagation, and coalescence). The plastic work component is computed from the plastic strain increment and the current stress state, while the damage component is implicitly accounted for through the reduction of elastic modulus in damaged zones. This definition follows the energy partitioning framework established by Zhou et al. [18] and is consistent with the thermodynamic formulation of rock failure processes.

### 3.3. Instability criterion for energy catastrophe during excavation of consequent slopes

The excavation process disrupts the original stress equilibrium within the slope, leading to a gradual rebalancing of the stress field over time. This transition inherently reduces slope stability. According to dissipative energy theory, slope instability fundamentally arises from the nonlinear transition of dissipative energy from a non-equilibrium to an equilibrium state over time [45–48].

By treating the slope as a holistic system under catastrophe theory, the evolution of total dissipative energy during excavation is modeled as a nonlinear state variable. The total dissipative energy Δ*U*_*d*_ is expressed as a time-dependent function:


$$\begin{array}{c} \Delta U_{d}=\sum_{u=1}^{N}\Delta U_{d}\left(t_{i}\right) \end{array}$$ (9)

where: *N*: Total number of slope elements at time *t*_*i*_. Δ*U*_*d*_(t): Incremental dissipative energy of elements at time *t*_*i*_.

Following Zhou et al. [18], let *ΔU*_*d*_(*t*_*i*_)=*f*(*d*), where *t*_*i*_ denotes energy evolution time during each excavation step, and *d* represents the time step in finite element simulations. A fifth-order Taylor expansion of Δ*U* yields:


$$\begin{array}{c} \Delta U_{d}\left(t_{i}\right)=f\left(d\right)=\alpha_{\text{0}}+\alpha_{\text{1}}d+\alpha_{\text{2}}d^{\text{2}}+\alpha_{\text{3}}d^{\text{3}}+\alpha_{\text{4}}d^{\text{4}}+\alpha_{\text{5}}d^{\text{5}} \end{array}$$ (10)

where coefficients $\alpha_{i}={\frac{\partial^{i}f}{\partial t^{i}f}|}_{t=0}$ are determined via MATLAB-based curve fitting of *ΔU*^*d*^(*t*_*i*_) versus *t*. Differentiating Formula (10) gives:


$$\begin{array}{c} \Delta {U_{d}}^{\prime }\left({\text{t}}_{i}\right)=f^{\prime }\left(t\right)=\alpha_{\text{1}}+2\alpha_{\text{2}}t+3\alpha_{\text{3}}t^{\text{2}}+4\alpha_{\text{4}}t^{\text{3}}+5\alpha_{\text{5}}t^{\text{4}} \end{array}$$ (11)

To render Formula (11) amenable to cusp catastrophe analysis, it is necessary to eliminate the quartic term and reduce the polynomial to a cubic form. This is achieved through the Tschirnhaus transformation [49], which introduces a change of variable t=m−n with $n=a_{\text{4}}/\text{5}a_{\text{5}}$. The transformation shifts the origin of the time coordinate, effectively removing the fourth-order term and yielding a canonical cubic polynomial. The detailed derivation is as follows:


$$\begin{array}{c} \left[\begin{array}{c} @l@b_{\text{0}}\\ b_{\text{1}}\\ b_{\text{2}}\\ b_{\text{4}} \end{array}\right]=\left[\begin{array}{ccccc} @ccccc@5n^{\text{4}} & -4n^{\text{4}} & 3n^{\text{4}} & -2n^{\text{4}} & 1\\ -20n^{\text{4}} & 12n^{\text{4}} & -6n^{\text{4}} & 2 & 0\\ 30n^{\text{4}} & -12n^{\text{4}} & 3 & 0 & 0\\ 5 & 0 & 0 & 0 & 0 \end{array}\right]\cdot \left[\begin{array}{c} @l@a_{\text{5}}\\ a_{\text{4}}\\ a_{\text{3}}\\ a_{\text{2}}\\ a_{\text{1}} \end{array}\right] \end{array}$$ (12)

Eliminate the cubic term of Formula (11). Available Formula (13);


$$\begin{array}{c} \Delta {U_{d}}^{\prime }\left({\text{k}}_{i}\right)=b_{\text{0}}+b_{\text{1}}m+b_{\text{2}}m^{\text{2}}+b_{\text{4}}m^{\text{4}} \end{array}$$ (13)

The coefficients a, b in Formula (12) are consolidated expressions of the original Taylor coefficients $a_{\text{1}}$ through $a_{\text{5}}$, As defined in Formula (14). These consolidated coefficients serve as the basis for the subsequent nondimensionalization and bifurcation set derivation.


$$\begin{array}{c} \left\{ \begin{array}{c} @l@b_{\text{0}}=5a_{\text{5}}n^{\text{4}}-4a_{\text{4}}n^{\text{3}}+3a_{\text{3}}n^{\text{2}}-2a_{\text{2}}n+a_{\text{1}}\\ b_{\text{1}}=-20a_{\text{5}}n^{\text{3}}+12a_{\text{4}}n^{\text{2}}-6a_{\text{3}}n+2a_{\text{2}}\\ b_{\text{2}}=30a_{\text{5}}n^{\text{2}}-12a_{\text{4}}n+3a_{\text{3}}\\ b_{\text{4}}=5a_{\text{5}} \end{array}\right. \end{array}$$ (14)

The mathematical transformations from Formulas (12) to (14) serve a dual purpose. First, the Tschirnhaus transformation t=m−n (with $n=a_{\text{4}}/\text{5}a_{\text{5}}$) eliminates the quartic term in the Taylor expansion, reducing the fifth-order polynomial to a canonical cubic form. It maps the complex energy evolution trajectory onto a reduced phase space where the system’s stability is governed by only two control variables (u and v), rather than multiple higher-order coefficients. Second, the subsequent nondimensionalization (Formula 15) removes the physical dimensions from the control variables, allowing the bifurcation set Δ to serve as a scale-invariant stability indicator.


$$\begin{array}{c} m=\sqrt[{4}]{\frac{\text{1}}{4\left|b_{\text{4}}\right|}}r \end{array}$$ (15)

And substituting into Formula (13), the potential function of the cusp catastrophe model is derived:


$$\begin{array}{c} U=\left\{ \begin{array}{c} @l@\frac{\text{1}}{\text{4}}r^{\text{4}}+\frac{b_{\text{2}}}{2\sqrt{\left|b_{\text{4}}\right|}}r^{\text{2}}+\frac{b_{\text{1}}}{\sqrt[{4}]{\sqrt{\left|b_{\text{4}}\right|}}}r+b_{\text{0}}\;(b_{\text{4}}>0)\\ -\frac{\text{1}}{\text{4}}r^{\text{4}}+\frac{b_{\text{2}}}{2\sqrt{\left|b_{\text{4}}\right|}}r^{\text{2}}+\frac{b_{\text{1}}}{\sqrt[{4}]{\sqrt{\left|b_{\text{4}}\right|}}}r+b_{\text{0}}\;(b_{\text{4}}<0) \end{array}\right. \end{array}$$ (16)

Where: *U*: Potential function. *r*: State variable. *u*, *v*: Control variables. *b*_*0*_: Constant term unrelated to catastrophe characteristics.

The equilibrium surface and singularity set are determined by:


$$\begin{array}{c} \begin{array}{c} u=\frac{b_{\text{2}}}{\sqrt{\left|b_{\text{4}}\right|}}\\ v=\frac{b_{\text{1}}}{\sqrt[{4}]{4\left|b_{\text{4}}\right|}} \end{array} \end{array}$$ (17)

According to the cusp catastrophe theory, *r* is the state variable, and the total potential function *U* is derived. The equilibrium surface of the qualitative energy dissipation analysis of the surrounding rock of the roadway is obtained as Formula (18).


$$\begin{array}{c} \frac{\partial U}{\partial r}=\left\{ \begin{array}{c} @l@r^{\text{3}}+ur+v=0\\ -r^{\text{3}}+ur+v=0 \end{array}\right. \end{array}$$ (18)

It can be seen from the catastrophe theory $\frac{\partial^{\text{2}}U}{\partial r^{\text{2}}}=0$ that the catastrophe point singularity set of the catastrophe theory satisfies Formula (19):


$$\begin{array}{c} \frac{\partial^{\text{2}}U}{\partial r^{\text{2}}}=\left\{ \begin{array}{c} @l@3r^{\text{2}}+u=0\\ -3r^{\text{2}}+u=0 \end{array}\right. \end{array}$$ (19)

The standard form of the bifurcation set equation of the cusp catastrophe theory model is Formula (20).


$$\begin{array}{c} \Delta =4m^{\text{3}}+27n^{\text{2}}=0 \end{array}$$ (20)

In the formula: *m*, *n* are dimensionless control variables.

The bifurcation set Δ of the potential function of the cusp catastrophe model of the surrounding rock of the roadway in the excavation of *k*_*i*_ m can be obtained by eliminating *r* in the simultaneous elimination of equations (19) and (20). Formula (21):


$$\begin{array}{c} \Delta =4u^{\text{3}}+27v^{\text{2}}=0 \end{array}$$ (21)

The bifurcation set Δ is rendered dimensionless through the nondimensionalization procedure in Formula (15), where the state variable r and control variables u and v are normalized by appropriate reference quantities. Specifically, $u=\frac{b_{\text{2}}}{\sqrt{\left|b_{\text{4}}\right|}},v=\frac{b_{\text{1}}}{\sqrt[{\text{4}}]{\text{4}\left|b_{\text{4}}\right|}}$ (with a, b defined in Formula 14) are ratios of coefficients with consistent physical dimensions, ensuring that $\Delta =\text{4}u^{\text{3}}+\text{2}\text{7}v^{\text{2}}$ is a pure number. This dimensionless nature allows Δ to serve as a universal stability criterion independent of the absolute scale of energy, facilitating direct comparison across different slopes and excavation conditions.

According to the catastrophe theory, the stability of the slope during the excavation of ki meters can be judged by the change of the bifurcation set Δ, which is the criterion for the energy dissipation mutation of the slope rock mass: when Δ < 0, the slope system crosses the bifurcation point set, that is, the total dissipation energy of the slope changes abruptly, and the slope is in an unstable state. When Δ = 0, the slope is in a critical instability state.

From the above content, it can be concluded that only when the bifurcation set equation of the slope Δ < 0, the slope will fail after the kith excavation, so Δ < 0 is used as the criterion for the sudden change of dissipation energy after the kith excavation of the slope.

## 4. Numerical simulation analysis

### 4.1. Numerical model

**Fig 4:**
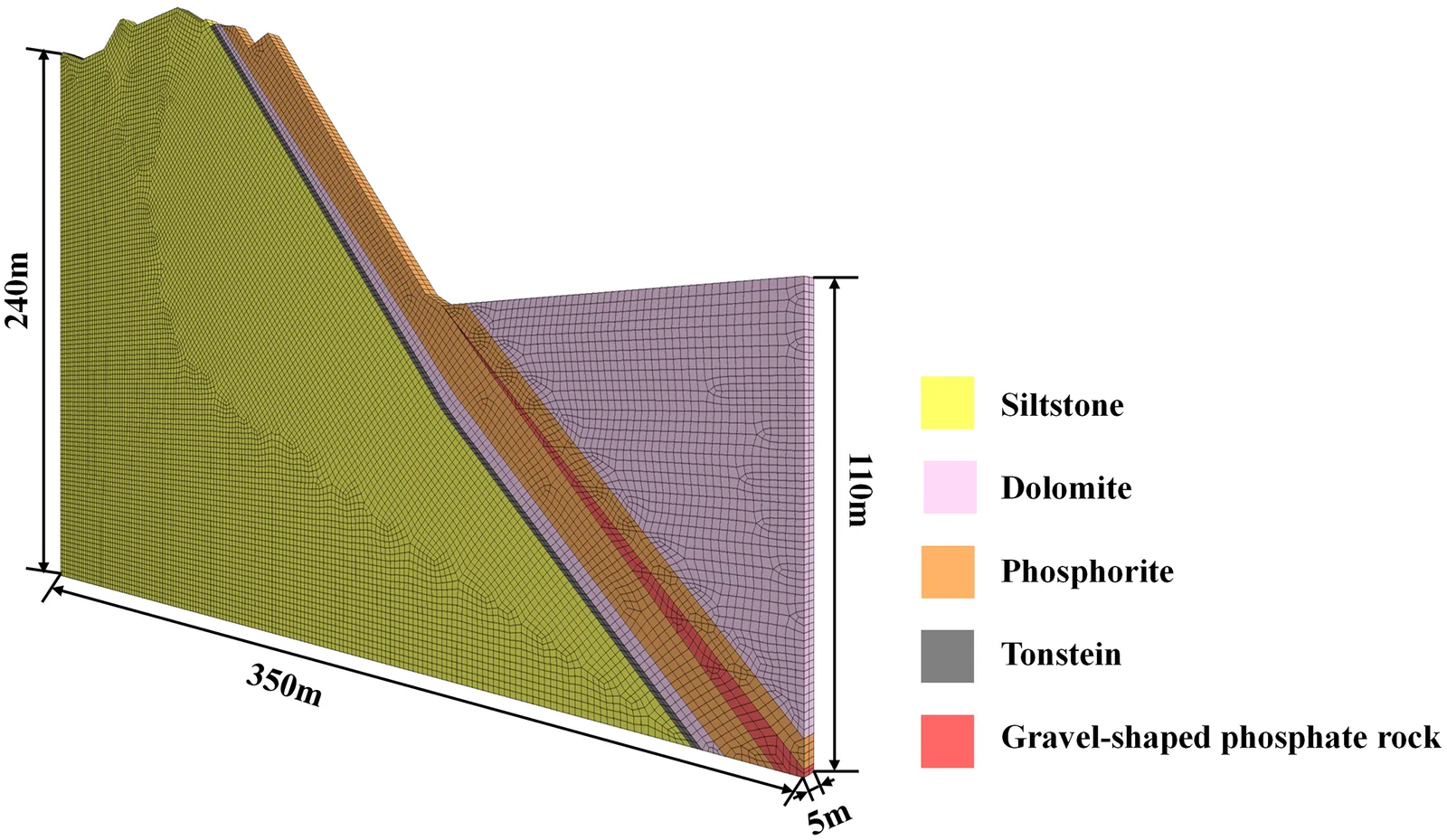
Numerical calculation model.

Based on the geological conditions of a certain phosphate mine in Guizhou Province, A numerical model with dimensions of 350 m (length) × 240 m (height) × 5 m (thickness) was constructed using Rhino software and imported into FLAC3D (Fig 4). Although the thickness is limited to 5 m, the model is configured as a three-dimensional representation to capture the out-of-plane stress effects and to enable the calculation of volumetric dissipative energy, which requires 3D integration over the element volumes. The thickness direction (z-axis) is assigned roller boundary conditions (i.e., $u_{z}=\text{0}$ at the front and rear faces), effectively imposing a plane-strain condition in the out-of-plane direction. This setup is consistent with the plane-strain assumption commonly adopted in slope stability analysis for long slopes, while still leveraging FLAC3D’s capability to compute 3D energy variables. The choice of 5 m thickness represents a unit slice that is sufficiently representative of the slope’s cross-sectional behavior, and the results are normalized per unit thickness for practical interpretation.

**Fig 5:**
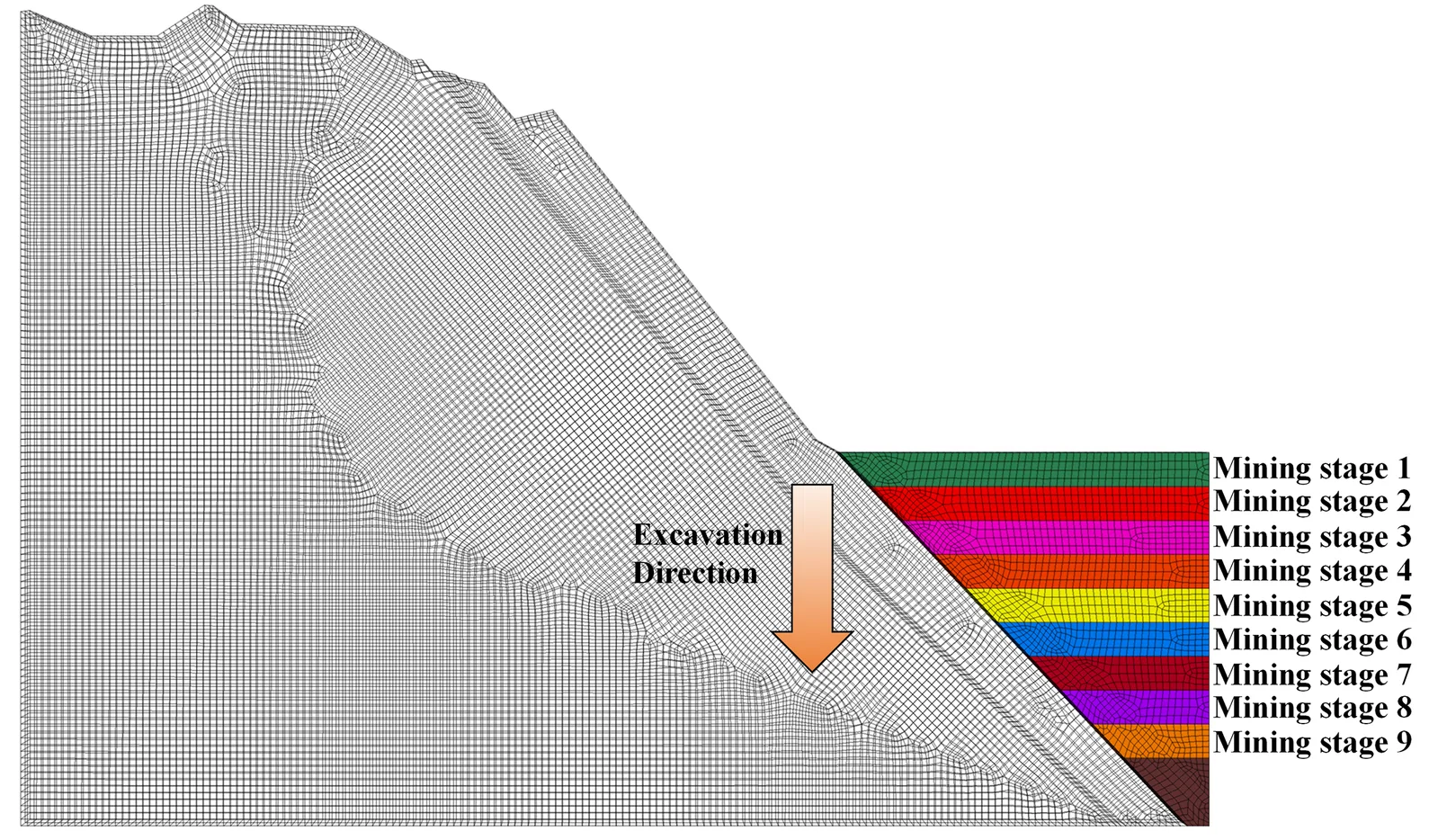
Schematic diagram of mining stages.

The model comprises 35,329 mesh elements and 48,110 nodes. Fixed constraints were applied to the bottom and side boundaries, while the top boundary remained unconstrained. To account for the bedding-plane anisotropy inherent in consequent slopes, the numerical model adopts a layer-wise property assignment approach: each element is assigned mechanical parameters (elastic modulus, Poisson‘s ratio, cohesion, and friction angle) according to its lithological layer as listed in Table 1. This layer-specific parameterization implicitly captures the first-order effects of bedding-plane anisotropy on stress redistribution and energy dissipation during excavation, without requiring a fully anisotropic constitutive model. The Mohr-Coulomb yield criterion was adopted for calculations. A clay interlayer within 20 m depth below the lowest platform at the slope crest of the typical cross-section was considered. Without slope cutting, unloading, or engineering reinforcement, the stability of the western wing floor slope was analyzed for each 10 m excavation stage, starting from the current pit bottom elevation [50–53]. The mining stages are illustrated in Fig 5.

### 4.2. Energy catastrophe evolution characteristics

**Fig 6:**
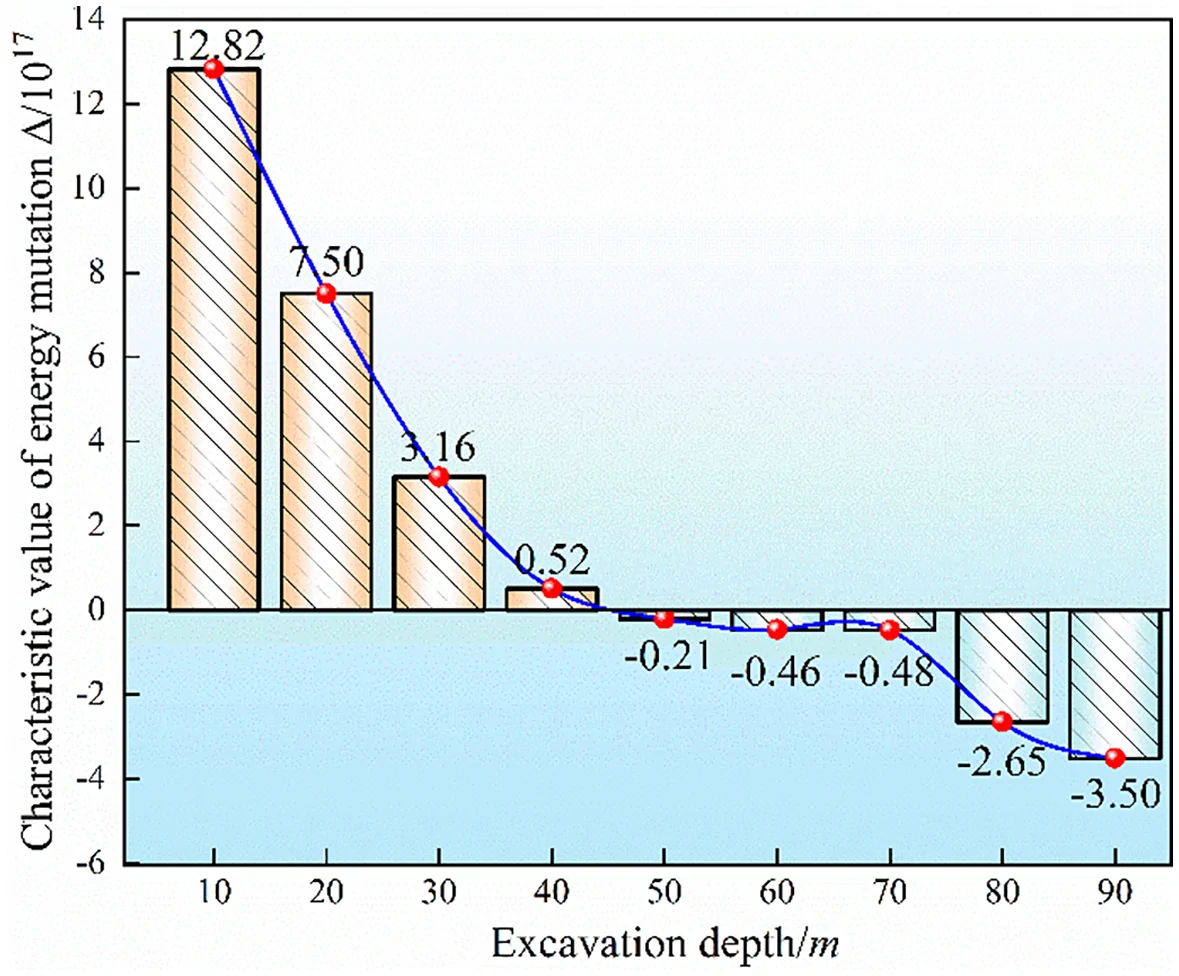
Catastrophic characteristic values (Δ) of slope energy.

The total dissipative energy Formula (21) was programmed into FLAC3D using its FISH language scripting function. This enabled the simulation of dissipative energy evolution during excavation and data exportation. The incremental total dissipative energy after each excavation step was calculated, and the catastrophic characteristic value Δ was derived based on Formula (21) [54]. Results are shown in Fig 6.

When the slope was excavated from 10 m to 40 m, Δ remained positive, indicating stable conditions. However, the gradual decrease in Δ values suggested progressive stability degradation. At 40 m excavation depth, Δ became negative, marking the transition from stable to unstable states. Further deepening of excavation intensified instability, as evidenced by a continued decline in Δ.

**Fig 7:**
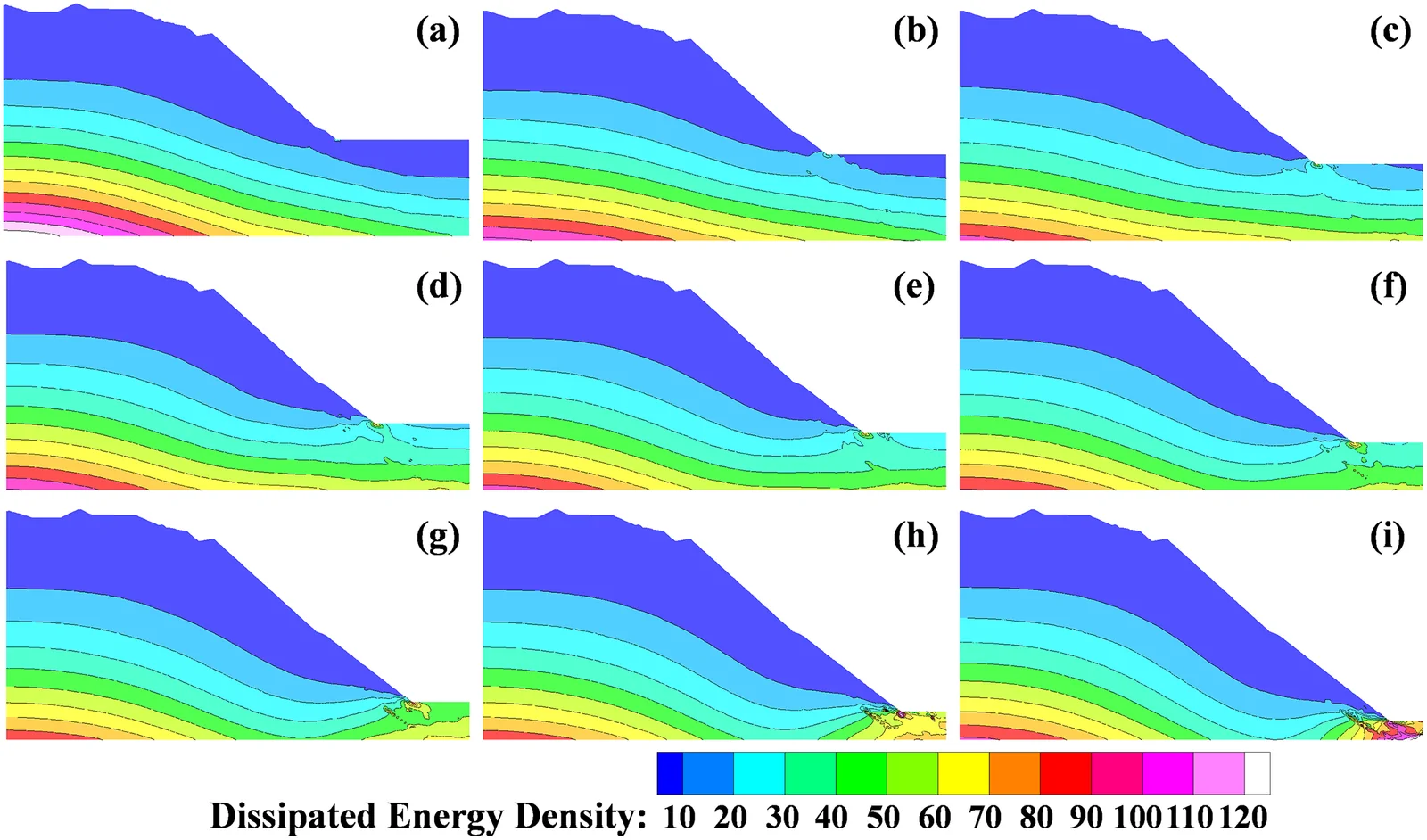
Contour maps of dissipative energy density: (a) 10 m, (b) 20 m, (c) 30 m, (d) 40 m, (e) 50 m, (f) 60 m, (g) 70 m, (h) 80 m, (i) 90 m excavation.

To systematically analyze dissipative energy evolution, energy density distributions after each excavation step were extracted and visualized as contour maps (Fig 7).

It can be seen from the diagram that with the excavation of the slope, the dissipated energy density at the slope surface is basically unchanged, indicating that the deformation degree of the slope is small and no damage occurs with the excavation of the slope. The dissipation energy density at the slope angle gradually increases, indicating that with the increase of excavation depth, the deformation at the slope angle gradually increases, and the degree of damage gradually increases.

It is shown in Fig 7 that when the slope is excavated to 10 m, the dissipation energy density at the foot of the slope is small, and the slope deformation is small, and no damage occurs. When the slope is excavated from 20 m to 40 m, the dissipation energy density at the toe of the slope gradually increases, but the dissipation energy density is still the energy limit that the rock mass at the toe of the slope can bear, so the rock mass at the toe of the slope still has certain stability. When the slope is excavated to 50 m, the dissipated energy density at the toe of the slope reaches the energy limit that the rock mass can bear, and the toe of the slope is destroyed. Currently, the slope changes from a stable state to an unstable state. Since then, with the increase of slope excavation depth, the degree of slope failure has gradually deepened.

### 4.3. Evolution characteristics of plastic zone

**Fig 8:**
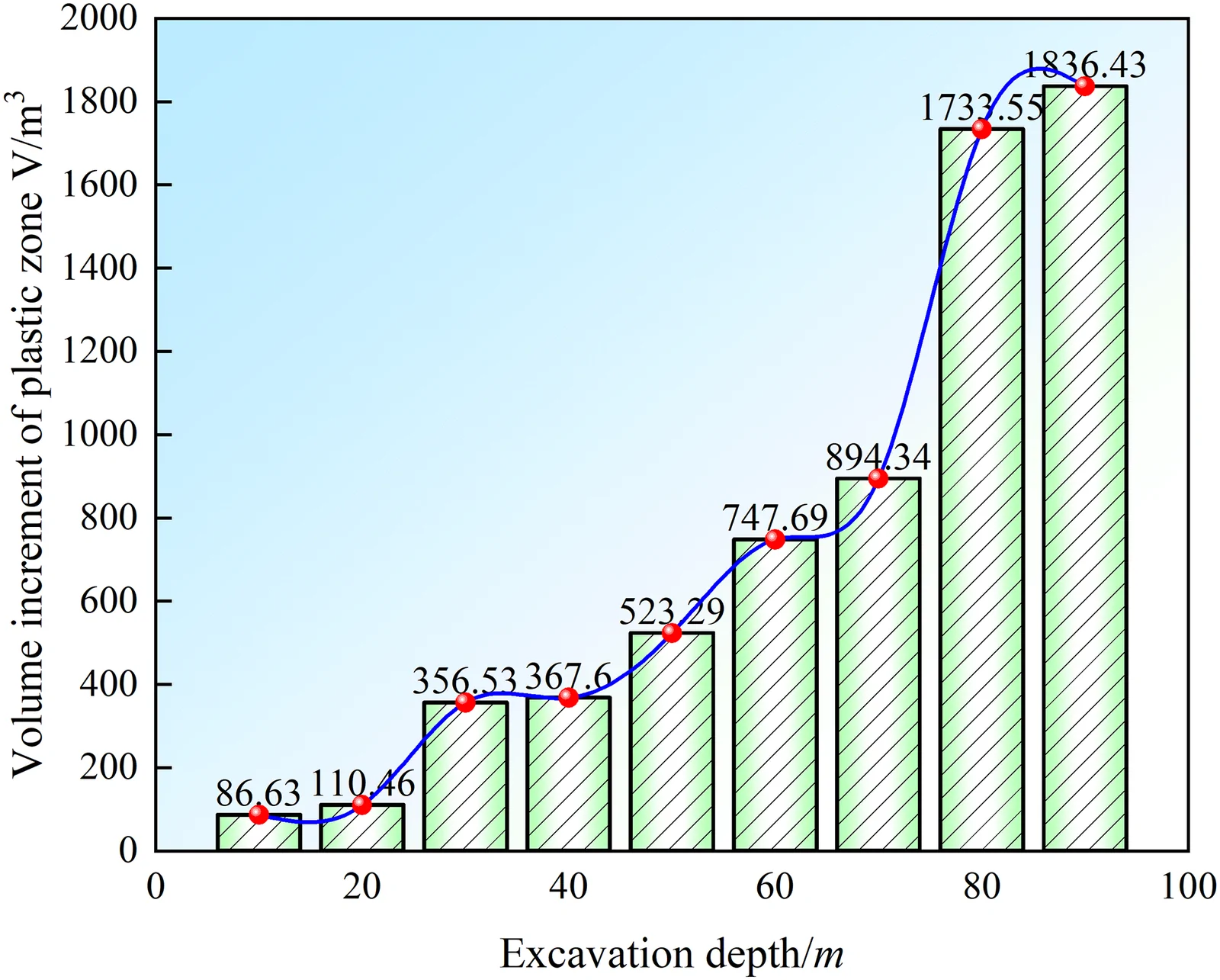
Volume increment of plastic zone.

**Fig 9:**
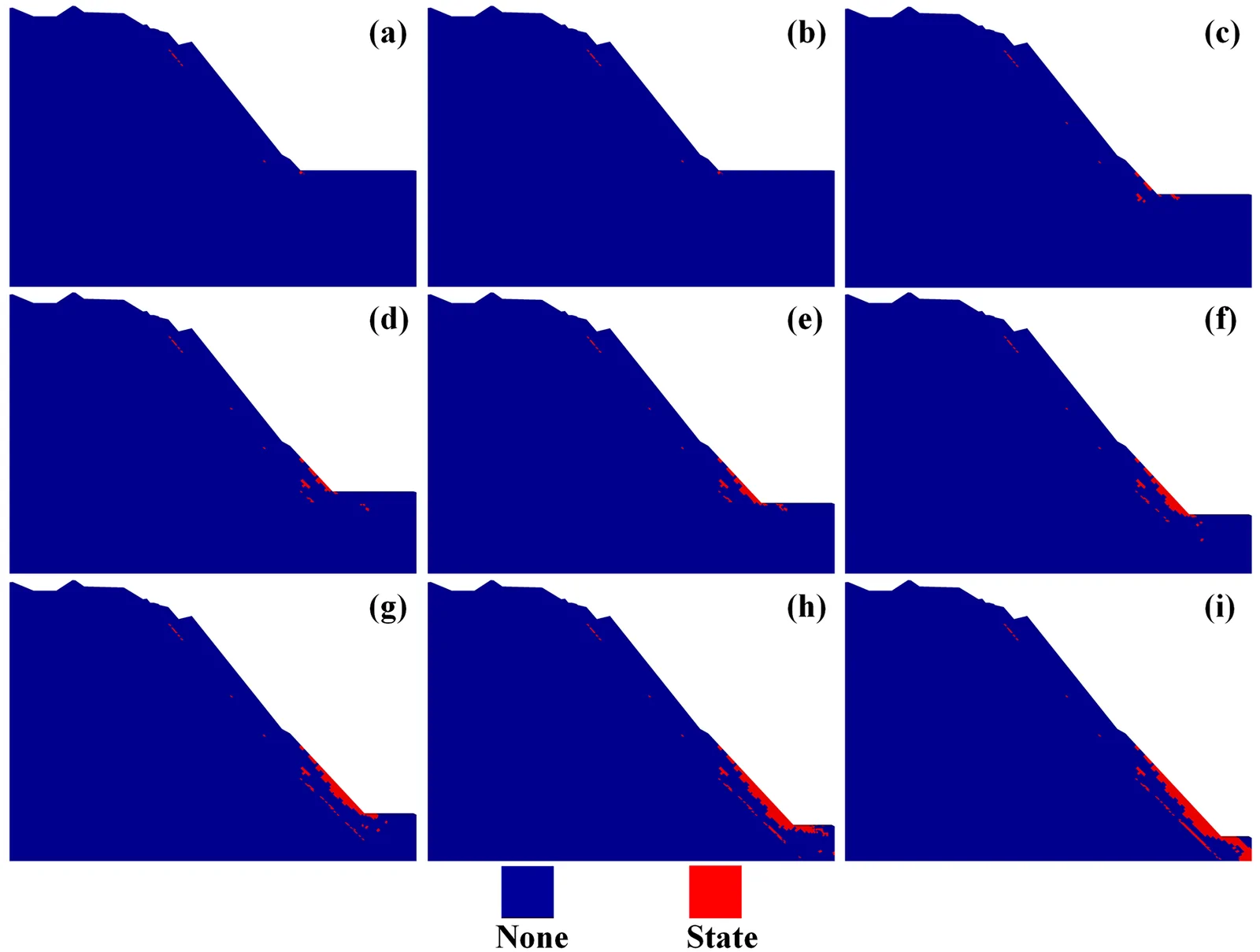
Cloud picture of plastic zone: (a) 10 m, (b) 20 m, (c) 30 m, (d) 40 m, (e) 50 m, (f) 60 m, (g) 70 m, (h) 80 m, (i) 90 m excavation.

In order to compare and analyze the evolution characteristics of slope stability in the process of slope excavation, the volume of plastic zone of slope in each mining stage is extracted, and the variation curve of plastic zone volume with mining depth is drawn as shown in Fig 8, and the distribution cloud map of plastic zone of slope in each mining stage is drawn as shown in Fig 9.

It can be seen from Fig 8 and Fig 9 that when the slope is excavated from 10 m to 20 m, there is a certain plastic zone on the slope surface, which does not penetrate the whole slope, but the volume increment of the plastic zone is less than 200 m^3^, indicating that the slope stability is high. When the slope is excavated to 30m and 40m, the plastic zone of the slope increases significantly, and the plastic zone begins to appear at the toe of the slope, indicating that the toe of the slope begins to fail, and the volume of the plastic zone of the slope is about 400m^3^, indicating that the slope still has certain stability at this time. After that, when the slope is excavated from 50 m to 90 m, the volume increment of the plastic zone of the slope increases gradually, and the plastic zone at the foot of the slope penetrates downward, indicating that the damage degree at the foot of the slope gradually deepens when the slope is excavated from 50 m to 90 m, and the slope changes from a stable state to an unstable state, which is the same as the above theoretical analysis results.

## 5. Slope stabilization measures and field applications

### 5.1. Mechanisms of slope instability and stabilization principles

(1) Mechanisms of Slope Instability

Based on the theoretical analysis of a certain phosphorus mine slope in Guizhou Province, combined with its geological characteristics and engineering conditions, and in accordance with the energy theory, the main factors causing the instability of the slope are summarized as follows:

a. Insufficient Energy-Bearing Capacity of Slope Rock and Soil Masses.

The phosphate rock on the slope exhibits weak lithological properties. During excavation, the disturbance caused by the process leads to significant energy accumulation within the slope. Due to the low energy-bearing limit of the phosphate rock, the accumulated energy under excavation disturbance can easily reach or exceed the critical threshold, resulting in failure.

b. Lack of Effective Support Structures.

Effective support structures can absorb the energy generated during excavation, reducing the energy accumulated in the slope rock and soil masses. This, in turn, minimizes the dissipated energy available for deformation and failure.

(2) Principles of Slope Stabilization

During excavation, the energy accumulated at the slope toe gradually increases. According to the principle of energy conservation, instability occurs when the accumulated energy at the slope toe exceeds the energy-bearing capacity of the rock mass. Based on the instability mechanism of a certain phosphate mine, as well as the geological and engineering conditions, the following stability principles are proposed:

a. Optimizing Slope Geometry.

Energy accumulation in rock masses is primarily caused by discontinuities, leading to localized energy concentration. To address this issue at the slope toe, the slope angle at the toe should be reduced during excavation. This modification lowers the sliding force of the rock and soil masses, distributing the accumulated energy to deeper rock layers and thereby reducing the risk of instability.

b. Enhancing Support Structures.

To resolve the lack of effective support, pre-stressed anchor cables should be applied to reinforce the slope face and toe during excavation. This approach establishes effective support structures capable of absorbing dissipated energy generated during the excavation process, ensuring slope stability.

### 5.2. Slope stabilization measures

**Fig 10:**
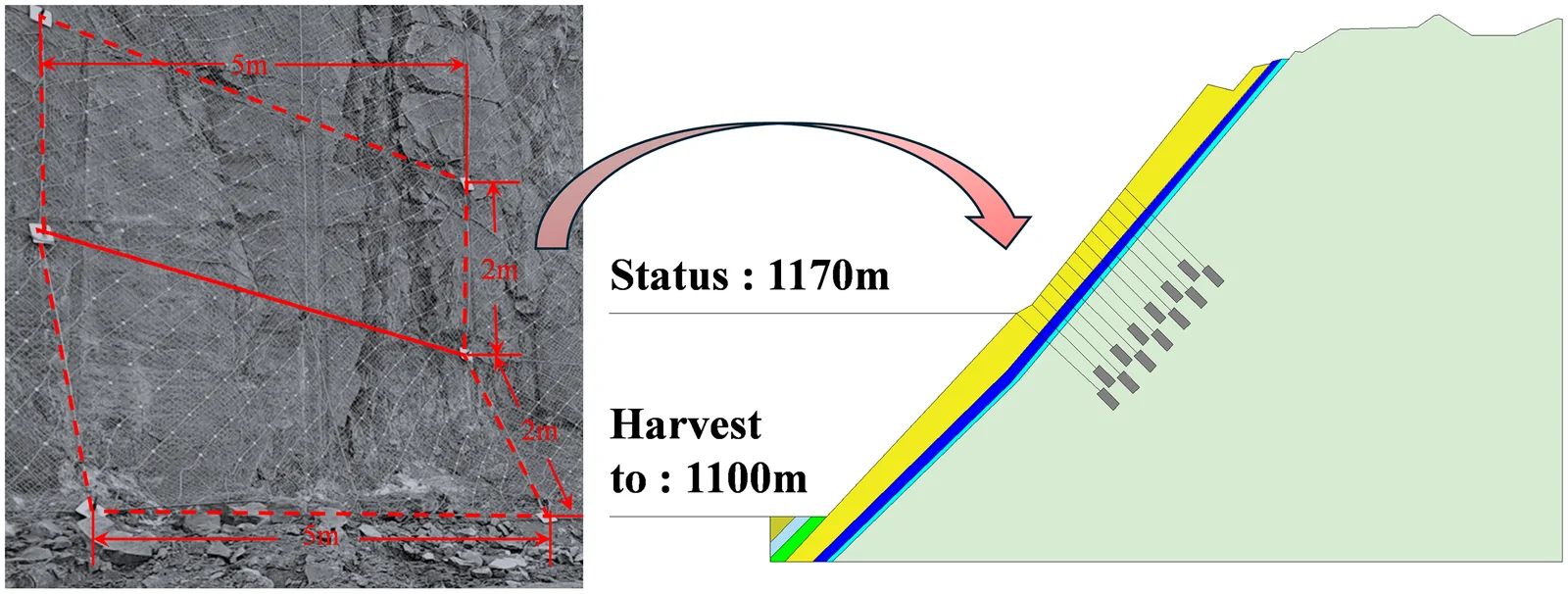
Construction diagram of pre-stressed anchor cables.

Based on the geological characteristics and energy evolution patterns of a bedding slope of a phosphate mine in Guizhou Province, the research team proposed and implemented a graded dynamic support system to control stability during mining. The stabilization measures prioritized bolt reinforcement to initially stabilize the western wing floor slope, ensuring an angle ≤40° between the slope and horizontal plane. Starting from the current pit bottom elevation of 1170 m, layered pre-stressed anchor cables were subsequently installed. Each anchor cable consisted of 10 bundles of Φ15.2 mm high-strength steel strands, with a single-strand locking force of 1300 kN. The cables were arranged on the slope surface at spacings of 3 m × 3 m or 5 m × 2 m, adhering to a design requirement of vertical projected load ≤10 m². During construction, the “excavate-and-anchor” principle was strictly followed: after each 10 m of layered excavation, anchor cables were immediately installed, tensioned, and locked, forming a dynamic closed-loop process of “excavation-support-monitoring” (Fig 10).

### 5.3. Industrial-Scale Validation

**Fig 11:**
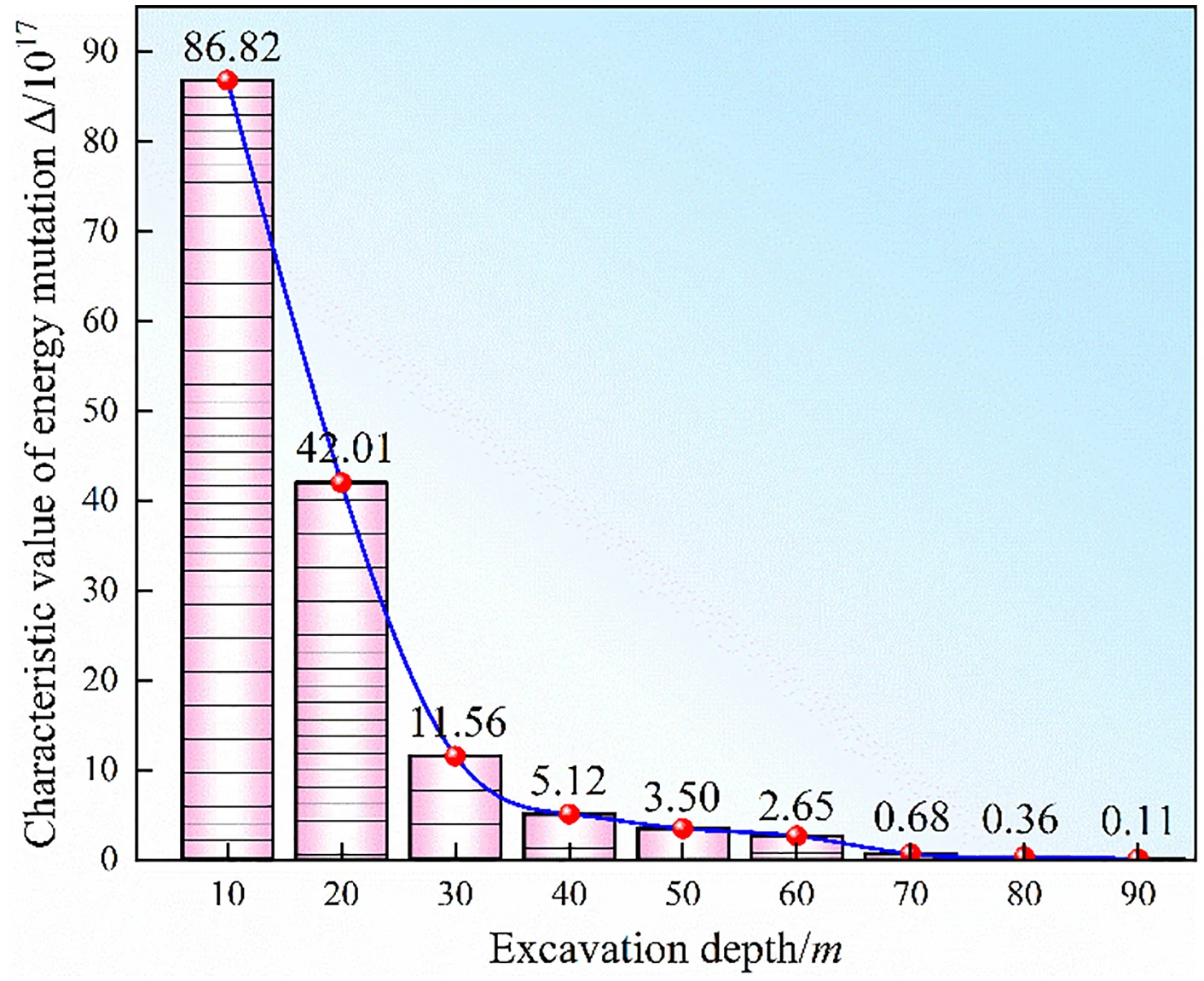
Post-support energy mutation characteristic values of the slope.

Through FLAC3D numerical simulations and field industrial-scale trials, the post-support energy mutation characteristic value (Δ) of the slope exhibited a negative correlation with excavation depth but remained within a stable range (Δ > 0) (Fig 11).

As shown in Fig 11, the energy mutation characteristic value decreased from 86.82 × 10¹⁷ (at 10 m excavation depth) to 0.11 × 10¹⁷ (at 90 m excavation depth), representing a reduction of 99.87%. However, Δ remained consistently above zero, indicating that the support structures effectively absorbed dissipated energy generated during excavation, thereby suppressing abrupt rock mass instability and ensuring long-term slope stability under deep mining conditions.

## 6. Conclusions

This study establishes an energy-based catastrophic instability criterion specifically tailored for excavated consequent slopes, extending the general cusp catastrophe framework of Zhou et al. [18] in three key aspects. First, by incorporating bedding-plane sliding and weak interlayer deformation into the dissipative energy formulation, our criterion captures the anisotropic failure characteristics that are absent in homogeneous slope models. Second, the proposed stability indicator Δ is validated against multi-stage field excavation data from the Wengfu Phosphate Mine, providing field corroboration beyond numerical simulations. Third, the criterion is translated into a gradient-anchoring reinforcement strategy informed by the spatial distribution of energy precursors, offering a direct engineering application that was not addressed in previous theoretical formulations. The main conclusions are summarized as follows:

(1) Energy-Based Instability Analysis:

By applying energy principles, this study revealed the intrinsic energy conversion mechanisms during slope excavation and derived a computational formula for the total dissipated energy. Integrating the cusp catastrophe theory, an energy mutation criterion was established to evaluate slope instability, with the mutation characteristic value (Δ) serving as a quantitative indicator of instability states.

(2) Numerical and Field Validation:

Using the FLAC3D software embedded with FISH language, the energy evolution and instability characteristics of a phosphate mine in Guizhou Province were analyzed based on the energy mutation criterion. The numerical simulation results indicated that there was a slope instability phenomenon at the excavation depth of 50 meters. By comparing the predicted results of this criterion with the actual observed development of the plastic zone, the validity of the criterion was further verified.

(3) Practical Stabilization Strategy:

Based on energy evolution laws and mutation characteristics, the energy-driven instability mechanism was elucidated, leading to the formulation of stability control principles. Guided by these principles, a pre-stressed anchor cable reinforcement system was designed and applied onsite. Post-implementation monitoring confirmed that the stabilized slope maintained long-term equilibrium under deep excavation conditions.
